# Multimodal perspectives on dental anxiety etiology and technologies

**DOI:** 10.6026/973206300220553

**Published:** 2026-01-31

**Authors:** Sana Khan, Vishwa Patel, Osiris Toledo Calixto, Akshitha Pathipati, Sai Viswasri Immameni, Asna Masood

**Affiliations:** 1 Department of Dental Surgery, M.A Rangoonwala College Of Dental Science & Research Centre, Pune, Maharashtra, India; 2 Department of Dental Surgery, Karnavati School of Dentistry, Gujarat University, Gandhinagar, Gujarat, India; 3 Department of Stomatology, Benemerita Universidad Autonoma De Puebla, Puebla, Mexico; 4 Department of dental surgery, Government Dental College and Hospital, Hyderabad, Telangana, India; 5 Department of Dental Surgery, Gitam Dental College & Hospital, Visakhapatnam, Andhra Pradesh, India; 6 Department of Dental Surgery, Panineeya Institute of Dental Sciences and Research Centre, Hyderabad, Telangana, India

**Keywords:** Dental anxiety, oral health, dental anxiety management, anxiety assessment tools, technological advancements

## Abstract

Dental anxiety is a complex condition that negatively impacts oral health, quality of life and dental care efficiency, often arising from psychological, physiological and early negative experiences that extend into adulthood. Therefore, it is of interest to assess its severity and identify triggers, tools such as the dental anxiety scale (DAS), modified dental anxiety scale (MDAS), dental fear scale (DFS) and visual analog scale (VAS). Children are often evaluated using the venham picture test (VPT) and facial image scale (FIS). Management involves behavioral and psychological methods, including rapport building, cognitive behavioral therapy and relaxation techniques. Pharmacological options like nitrous oxide and sedation provide relief. Oral benzodiazepines help patients remain calm during clinical procedures. Additionally, emerging technologies like virtual reality, augmented reality, audiovisual distraction and biofeedback have shown promising results. Tailored, multimodal plans ensure the best outcomes for every individual. Integrating these tools leads to better cooperation and long-term health.

## Background:

Dental anxiety is one of the most common conditions present among the masses globally. It is this fear that makes individuals avoid seeking dental treatment which results in a deteriorated oral health-related quality of life [1]. Odontophobia (dental fear) is a "unique phobia with special psychosomatic components that impact on the dental health of the odontophobic persons" [2]. Dental anxiety fosters a multifaceted cycle of appointment avoidance and declining oral health, though the precise causality remains unknown [3]. Lack of timely attention to an individual's teeth can cause issues such as carious teeth, periodontitis and, consequently, the loss of natural teeth [4]. This behavior isn't limited to the dentist's office; it can also affect daily life by causing changes in eating behavior, oral hygiene and social interactions, as well as leading to self-medication, depression, aggression and sleep problems [1]. An understanding of the factors underlying the etiology and maintenance of dental anxiety may assist dentists and researchers in developing interventions designed to reduce dental avoidance behaviors [5]. Understanding the extremely complex psychology of dental fear is essential in the prevention and treatment of dental anxiety, fear and phobias [6]. Therefore, it is of interest to show the impact and management of dental anxiety.

## The etiology of dental anxiety:

Dental anxiety is a multifactorial problem with an unclear origin [7]. In the evolutionary perspective, fear is a key component of survival. Generally, fear is manifested as a response to immediate danger, while anxiety is a reaction to potential danger. In a dental context, dental fear is a reaction to something interpreted as a threat and dental anxiety is a state of apprehension. Several studies report that patients with dental anxiety have negative thoughts about dental treatments, which increase anxiety and trigger physiological reactions [8]. Understanding and promptly addressing the factors that can trigger anxiety is paramount to preventing more serious stages and providing tailored treatment [7]. A painful dental visit during childhood can have long-lasting effects that persist through adulthood. In children, elevated stress about dental visits is associated with a higher incidence of dental caries and a notable decrease in tooth brushing. Treating anxious patients is a stressful situation not only for the patients themselves but also for the dentist, due to reduced cooperation, extended appointment times and the overuse of resources. New studies have found a strong relationship between psychological disorders and dental anxiety. People who suffer from a particular phobia could be predisposed to anxiety in general [9].

## Impact of dental anxiety:

Dental anxiety creates a vicious cycle of avoidance and deterioration, significantly impacting a person's oral health and quality of life. This fear leads to poor oral hygiene and the avoidance of necessary dental care, allowing minor issues to worsen. This cycle not only perpetuates the patient's fear and shame but also negatively affects dental professionals.

## The cycle of dental anxiety:

Dental anxiety creates a cycle where fear of dental procedures leads to avoidance of routine and necessary dental visits. This avoidance, in turn, allows oral health to decline, leading to more serious problems that require complex and potentially painful emergency treatments. These more invasive procedures then justify and reinforce the person's initial fear, continuing the cycle [10].

## Here is how the cycle unfolds:

A person with dental anxiety anticipates pain or a negative experience at the dentist, causing them to delay or cancel appointments [11]. Without regular check-ups and cleanings, their oral health deteriorates and minor issues like cavities and periodontal diseases worsen [12]. This leads to poorer oral hygiene and a higher need for dental intervention [13]. The individual often experiences shame and fear of clinical reprimand, creating a psychological feedback loop that reinforces dental care avoidance [11]. When the individual finally seeks care, it's often for a dental emergency, which may require complicated and potentially painful treatments [12]. The negative experience of the emergency procedure validates the person's fear, making them even more likely to avoid future dental visits and restart the cycle [13].

## Impact on oral health and quality of life:

Dental anxiety has a significant impact on oral health and overall well-being. Studies have shown that people with high dental anxiety tend to have poorer oral hygiene and a higher number of decayed, missing and filled teeth. Anxious individuals often neglect daily hygiene and preventive visits, precluding early clinical intervention and accelerating dental deterioration [14]. Poor oral health, which is often a consequence of dental anxiety, can lead to social embarrassment and affect a person's psychological state. This can impact on their confidence and social interactions, ultimately diminishing their overall quality of life [15].

## Challenges for dentists:

Dental anxiety is not only a problem for patients; it also creates significant challenges for dental professionals. A fearful patient can be uncooperative, leading to longer treatment times and increasing the risk of misdiagnosis or mistreatment, such as an inaccurate tooth vitality analysis. Working with an anxious patient can induce clinician stress, potentially damaging communication and fostering blame-centric dynamics that impair the relationship [16]. Despite significant advancements in oral healthcare and hygiene practices over the past few decades, many people with dental anxiety have not benefited from these developments. This highlights the urgent need to address the psychological barriers that prevent individuals from receiving the care they need [14].

## Assessment and measurement tools:

The importance of evaluating dental anxiety before treatment has led to the development of a variety of instruments to identify and score dental anxiety [17]. The most widely used dental assessment instruments appear below:

## Dental anxiety scale (DAS):

The first is corah's dental anxiety scale (DAS), created in 1963. It is the most commonly used dental scale and is available in over 20 languages. This scale assesses dental anxiety related to four scenarios: expecting to see a dentist the following day, waiting in the waiting area, having teeth polished or scaled and getting a restoration. For each scenario, the patient chooses one of the five options that most accurately represent their current condition and each option is given a score of [1, 2, 3, 4- 5]. The total score is calculated by adding scores given to each scenario and ranges from [4, 5, 6, 7, 8, 9, 10, 11, 12, 13, 14, 15, 16, 17, 18, 19-20]. The scores are interpreted as follows: score [4-5] non-anxious, [6, 7, 8, 9- 10] slightly anxious, [11, 12, 13, 14-15] fairly anxious and [16, 17, 18, 19-20] extremely anxious. Studies have demonstrated that the results produced by das are highly stable and reliable [17].

## Modified dental anxiety scale (MDAS):

Despite DAS's widespread acceptance, it failed to account for local anesthesia/injection fear, which has been shown to be a major contributing factor to dental anxiety. As a result, the modified dental anxiety scale (MDAS) now includes a new scenario related to local anesthesia. Therefore, the overall score ranges from 5 to 25 serious dental anxieties and the need for therapeutic care are indicated by a score of 19 or higher. This measurement tool has been considered relatively easy to use and studies have demonstrated good consistency and validity of the results [18].

## Dental fear scale (DFS):

The second most often used measure for assessing dental anxiety is the dental fear scale (DFS), which was developed by kleinknecht in 1973. There are now 20 items on the scale, compared to the original 27. Of all the 20 items, 12 items measure fear of particular dental tools or stimuli, 5 items measure physiological responses, 2 items measure avoidance behavior and 1 item measures general fear of dental care. DFS does not offer a dental anxiety score, unlike other assessment tools. Instead, it helps dentists evaluate and identify the underlying causes of a patient's dental fear, making it a practical aid in dental settings [19].

## Visual analog scale (VAS):

The visual analog scale (VAS) is a simple and time-efficient tool and typically takes less than one minute to complete, making it well-suited to use in epidemiological and clinical studies to assess dental anxiety [20]. VAS is usually presented as a 10 cm long horizontal line, with endpoints marked as "not anxious" and "extremely anxious." Patients mark a point on the line to represent their present level of anxiety. The distance from the "not anxious" end to the patient's mark represents their level of anxiety. Research has demonstrated that vas is a reliable metric that is similar to MDAS [21].

## Venham picture test (VPT):

Eight cards with two images - an anxious figure and a non-anxious figure make up the venham picture test. The child will be asked to choose the figure that most accurately depicts their present situation [18]. Each time the child chooses an anxious face, a score of one is assigned. As a result, the overall score goes from 0 to 8, where a higher number denotes more dental anxiety. This scale is simple and ideal for assessing dental anxiety in young children, as they typically have limited cognitive and communication skills [22].

## Facial image scale (FAS):

The facial image scale is a simple tool designed to measure dental anxiety in very young children. Children select one of five representative faces to indicate their current state, allowing clinicians to interpret their level of anxiety. Though FAS is very simple and easy to use, it doesn't provide much information on the type or reason for anxiety [20].

## Psychological and behavioral management: 

Dental anxiety is a prevalent concern affecting both pediatric and adult patients. To address this issue, a range of behavioral and psychological interventions have been developed, aiming to reduce fear and improve patient cooperation. Evidence from recent literature highlights that rapport, communication and structured therapies effectively reduce anxiety, either independently or alongside sedation [23].

## Behavioral management techniques:

Behavioral interventions focus on modifying patient responses to dental care through structured, practical techniques. Rapport building and clear communication are central to this approach, as they help establish trust and reduce uncertainty and fear. One of the widely used methods is the tell-show-do technique, a particularly effective technique where the dental procedure is first explained (tell), then demonstrated (show) and finally performed (do). This approach reduces fear of the unknown and is especially effective in pediatric patients [24]. Other techniques, such as positive reinforcement and modeling promote cooperation by rewarding desirable behaviors and providing successful peer examples to observe. Audiovisual distraction, such as music or video, has shown measurable benefits in redirecting attention away from the procedure and lowering perceived anxiety [25].

## Psychological management techniques:

Psychological interventions address the cognitive and emotional processes underlying dental anxiety. Among these, cognitive behavioral therapy (CBT) is one of the most widely studied and effective methods. It aims to restructure negative thought patterns related to dental treatment and promote adaptive coping mechanisms. CBT has been shown to be effective in both short-term symptom relief and long- term behavioral change. Similarly, rational emotive behavior therapy (REBT) REBT corrects irrational beliefs for balanced expectations, while relaxation techniques reduce physiological symptoms through controlled breathing and imagery [26]. These techniques are simple to use, easy to teach and effective in clinical settings [24]. In some cases, hypnosis may be used to induce a trance-like state that facilitates relaxation and reduces fear. However, its application is limited by the need for specialized training and variable patient receptivity [25]. Psych education is another technique that helps patients understand the nature of their anxiety and the dental procedures involved. This increased awareness often reduces fear of the unknown and improves patient cooperation. Music therapy, a non-invasive method, may enhance relaxation and promote a calmer clinical environment [27]. The dentist-patient relationship also serves as a psychological tool, where empathy, reassurance and individualized care significantly influence patient comfort and treatment acceptance. Importantly, the choice of appropriate interventions should consider both developmental and situational factors. For instance, in pediatric care, techniques like modeling and distraction are particularly effective, whereas adults may benefit more from cognitive strategies like CBT and REBT.

## Effectiveness and considerations:

The effectiveness of behavioral and psychological interventions depends on several factors, such as individual patient needs, severity of anxiety and consistency of application. Current literature emphasizes that a multimodal approach- combining behavioral, psychological and, when necessary, pharmacological methods yield the most reliable outcomes. Additionally, tailoring strategies to pediatric versus adult populations ensures optimal effectiveness, as coping mechanisms and cognitive capacities differ significantly across age groups [23].

## Pharmacological management:

## Inhalation sedation (nitrous oxide–oxygen):

Inhalation sedation with nitrous oxide is one of the most frequently utilized pharmacological approaches for managing dental anxiety. It offers rapid onset, easy titration and swift recovery, making it suitable for both adults and children. Evidence indicates that nitrous oxide is highly effective in reducing anxiety and improving patient cooperation during procedures [28]. Additionally, studies demonstrate that nitrous oxide has a favorable safety profile, with minimal adverse effects when administered according to established guidelines. Its minimal impact on cardiovascular and respiratory systems further reinforces its role as a first-line pharmacological option [29].

Each pharmacological approach has unique strengths and limitations and the choice depends on the patient's level of anxiety, medical history and the complexity of the dental procedure. Nitrous oxide is highly effective for mild to moderate anxiety and pediatric patients due to its safety and rapid recovery profile. Oral sedation is well-suited for moderate to severe anxiety during more complex or lengthy procedures, though it requires strict pre- and post-procedure precautions. IV sedation is the most effective in reducing severe anxiety and ensuring patient comfort but requires specialized training and carries greater risks. Evidence shows comparable efficacy between nitrous oxide and midazolam in children, allowing selection based on clinical setting and patient requirements [33]. Combination therapy, such as nitrous oxide with oral benzodiazepines, has also been shown to enhance anxiolysis in certain cases [30].

## Oral sedation (benzodiazepines and adjuncts:

Oral anxiolytics, most commonly benzodiazepines such as diazepam, lorazepam and midazolam, are another important tool in managing dental anxiety. These agents provide sedative, muscle relaxants and anxiolytic effects by binding to gamma-aminobutyric acid (GABA) receptors. Oral administration is convenient and generally well-accepted by patients. However, challenges include variability in absorption, delayed onset and prolonged recovery times, which can complicate post-treatment functioning. Despite these limitations, oral benzodiazepines remain useful for managing mild to moderate anxiety, especially for short and less complex dental treatments [30].

## Intravenous (IV) sedation:

Intravenous (IV) sedation provides a more controlled and predictable method of pharmacological management. Midazolam is the most widely used IV agent due to its rapid onset, short duration of action and amnesic properties. This technique ensures patient safety while preserving defensive reflexes by enabling the medicine to be titrated to the appropriate level of sedation. However, IV sedation requires specialized training, advanced monitoring and facilities, limiting its availability in general practice settings. Nevertheless, it is highly effective for patients with moderate to severe dental anxiety undergoing invasive procedures [31].

In extreme cases where other pharmacological or behavioral strategies fail, general anesthesia (GA) may be considered. GA eliminates anxiety entirely by rendering the patient unconscious, making it useful for highly phobic individuals, patients with special healthcare needs, or those requiring extensive surgical treatment. However, the risks associated with GA, including potential complications and the need for a hospital environment; restrict its use to cases where alternative methods are insufficient [32].

## Clinical considerations and comparisons:

Each pharmacological approach has unique strengths and limitations and the choice depends on the patient's level of anxiety, medical history and the complexity of the dental procedure. Nitrous oxide is highly effective for mild to moderate anxiety and pediatric patients due to its safety and rapid recovery profile. Oral sedation is well-suited for moderate to severe anxiety during more complex or lengthy procedures, though it requires strict pre- and post-procedure precautions. IV sedation is the most effective in reducing severe anxiety and ensuring patient comfort but requires specialized training and carries greater risks. Evidence shows comparable efficacy between nitrous oxide and midazolam in children, allowing selection based on clinical setting and patient requirements [33]. Combination therapy, such as nitrous oxide with oral benzodiazepines, has also been shown to enhance anxiolysis in certain cases [30].

## Technological management:

The introduction of technological innovations has presented varied approaches to reducing dental anxiety, which is widespread and tends to interfere with efficient oral healthcare provision. These technologies induce relaxation and cooperation by distracting patients and redirecting their cognitive attention toward more comfortable stimuli [34]. Virtual reality (VR) technology has proven to be an optimistic approach to dental anxiety and pain management without resorting to the use of drugs. VR systems make patients plunge into virtual realities, which take their focus off the dental setting and practice [35]. A randomized trial conducted in 2020 showed that brief waiting-room VR sessions significantly reduce both total and anticipatory dental anxiety in patients. Particularly, the level of total dental anxiety in the VR relaxation group was lower compared to the control group. It was also observed that the anticipatory dental anxiety reduced to a greater extent in the VR group in both men (β = -0.217, p < .026) and women (β = -0.498, p < .001). Overall modified dental anxiety scale (MDAS) score (VR vs. Control) was reduced in women (β = -1.08, p < .001) and so did treatment-related dental anxiety (β = -0.597, p = .011). These results support VR as a viable and useful approach in mitigating preoperative dental anxiety in those dental care facilities that are publicly accessible [34]. Augmented reality (AR) is another technological tool that has demonstrated potential in reducing dental anxiety, particularly among children. Unlike VR, AR maintains the patient's environmental awareness while providing digital enhancements to reduce clinical anxiety. The advantage of AR is the ability to enhance the real environment without full immersion, which can be more acceptable to some patients [37]. Another widely used technology is the audiovisual (AV) distraction methods, which includes the utilization of screens, videos and music in order to distract patients during dental procedures [38]. Physiological measures of anxiety, including heart rate, were significantly lower among children experiencing AV distraction during local anesthesia dental treatment as compared to control groups (p = 0.01). Moreover, AV distraction has been found to enhance cooperation in children and lessen the amount of anxiety perceived when undergoing dental treatment [39]. Biofeedback technology provides a more interactive approach, especially useful for managing acute anxiety during dental treatments. The recent study, particularly on the use of respiratory biofeedback in dental extractions, demonstrates the effectiveness, benefits and limitations of its use in reducing dental anxiety. Biofeedback provides real-time physiological data, enabling patients to consciously regulate unconscious responses like heart rate and breathing. This self-control, in relation to dental anxiety, gives patients a sense of empowerment to manage their anxiety reactions [40]. In summary, all these technologies, such as VR, AR, AV distraction and biofeedback, have their own benefits and ways of alleviating dental anxiety. VR and AR offer an immersive or enhanced reality experience, whereas AV tools provide access to a distraction and biofeedback equips patients with active coping skills. The body of evidence from the recent studies points to their efficacy in a wide range of patient groups, including children and adults, in the reduction of both subjective and objective indicators of anxiety [36, 39, 40].

## Conclusion:

Dental anxiety creates a cycle of avoidance and poor oral health, increasing treatment complexity and challenging both patients and clinicians. Effective management utilizes a robust toolkit of standardized scales, behavioral therapies and pharmacological options to address patient fear. Emerging technologies like VR, AR and biofeedback provide accessible non-pharmacological tools to foster distraction and patient self-management.
